# Classification of mushrooms based on AWPF-ResNet18

**DOI:** 10.1371/journal.pone.0346589

**Published:** 2026-04-16

**Authors:** Xinhai Zhao, Hanchen Lin, Yongmin Guo, Ning Sun

**Affiliations:** 1 College of Computer and Information Engineering, Tianjin Agricultural University, Tianjin, China; 2 College of Agronomy and Resources & Environment, Tianjin Agricultural University, Tianjin, China; Universidade Federal de Minas Gerais, BRAZIL

## Abstract

Edible mushrooms are widely enjoyed around the world for their unique flavor and rich nutrients. However, harvesting them carries the risk of accidentally picking poisonous varieties, leading to poisoning upon consumption. Edible mushrooms require classification during cultivation as well. This study presents AWPF-ResNet18, a ResNet18-based classification model that incorporates an Adaptive Window Pyramid Fusion (AWPF) module. AWPF performs dynamic multi-scale feature fusion and uses a Dynamic Swin Window module (DSW) with variable window sizes to refine downsampled features, thereby mitigating semantic information loss during downsampling. The model adaptively focused on targets of varying sizes within images, and achieved significant performance in the classification tasks with differentiate sizes. Experimental results show that incorporating the AWPF module improves the performance of the model over the original Residual Network 18 (ResNet18), with accuracy (Acc), macro precision (MP), macro F1-score (MF), and macro recall (MR) increasing by 2.5%, 7.5%, 5%, and 2%, respectively. Moreover, compared with current state-of-the-art classification models, the proposed design achieves varying degrees of improvement across relevant performance metrics. Multiple comparative experiments were conducted to validate its effectiveness. In summary, the AWPF-ResNet18 model demonstrates outstanding performance in edible mushroom classification tasks, offering an effective technical approach for the safe identification and categorization of mushrooms, and thus holds significant practical value.

## Introduction

Edible mushrooms are widely used in functional foods and dietary supplements because of their nutritional and medicinal value. They are rich in polysaccharides, including protein-bound polysaccharide K, which show bioactivities such as immunomodulatory, antitumor, antioxidant, and antiviral effects [1]. Most edible mushrooms belong to the phyla Basidiomycota and Ascomycota, and their edible fruiting bodies are macroscopic and can be harvested [2,3]. According to the *NY/T 749–2023 Safety Standards for Edible Fungi*, edible mushrooms should have desirable sensory qualities and must be non-toxic and safe for consumption [4]. Many edible species are collected from the wild, and some toxic species can be easily confused with edible ones based on morphology. For example, the edible *Cantharellus albovenosus* has a delicate, pale-yellow appearance, whereas the toxic *Omphalotus olearius* is morphologically similar despite its toxicity [5]. As a result, collecting wild mushrooms without reliable toxicity identification can lead to the accidental harvest and ingestion of highly toxic species, causing poisoning incidents [6]. Accurate classification and identification are also required during mushroom cultivation and production; however, traditional approaches rely largely on visual inspection and are prone to subjective errors [7]. In foraging and household settings, rapid photo-based identification of suspicious mushrooms using smartphones or portable devices may reduce the risk of accidental ingestion of toxic species. In industrial mushroom cultivation and sorting, accurate classification can enable automated grading, thereby reducing misclassification and labor costs. However, real-world data collected from both foraging and industrial settings are often limited in quantity and annotation. Moreover, many species are visually similar, and the class distribution is frequently highly imbalanced.

This study develops a lightweight yet robust model for edible mushroom classification. The model is designed to maintain reliable and balanced performance across classes under real-world conditions, including limited samples, visually confusing species, and class imbalance.

### Related work

Current edible mushroom classification relies on five main approaches: traditional morphological identification, spectroscopic analysis, molecular biological methods, physiological/biochemical assays, and machine-vision-based methods [8,9]. Traditional morphological identification is based on the morphology of fruiting bodies, hyphal characteristics, and microscopic structures. It is suitable for rapid on-site assessment and is relatively low-cost; however, it depends heavily on expert experience and is prone to misclassification among morphologically similar species. Spectroscopic approaches classify mushrooms by measuring differences in protein and amino acid profiles in the cap and by analyzing infrared absorption and reflectance patterns associated with molecular bonds. However, these measurements can be affected by moisture content and metabolites, and the required instruments are often expensive to operate and maintain [10]. Molecular methods identify species based on DNA sequence variation. A common strategy is to amplify and sequence the internal transcribed spacer (ITS) region and then compare the sequence with reference databases to achieve high classification accuracy [11]. However, fine-grained discrimination among species within the same genus can remain challenging, and these methods typically require dedicated laboratory facilities and trained personnel. Sequence characterized amplified region (SCAR) marker techniques can partially address this limitation, but their relatively high testing cost still restricts large-scale deployment [12]. Physiological and biochemical assays classify fungi by analyzing metabolites or environmental response indicators. These assays are relatively simple and low-cost, but they are time-consuming and can be sensitive to environmental conditions [13]. Machine vision methods acquire RGB images of mushrooms and analyze visual attributes such as color and texture to predict their taxonomic classes [14].

In recent years, image-based approaches for edible mushroom classification have gained popularity because they are relatively low-cost and efficient. In machine-vision-based studies, commonly used methods fall into two categories: conventional machine-learning approaches and deep-learning approaches that have rapidly advanced in recent years. Conventional machine-learning methods rely on hand-crafted image features and then use classifiers such as support vector machines (SVM) and random forests (RF) [15,16]. For typical edible mushroom classification tasks, random forests have been reported to achieve higher predictive accuracy than SVM-based models [17]. Compared with conventional machine-learning approaches, deep-learning models based on convolutional neural networks (CNNs) typically achieve higher classification accuracy [18]. However, recent studies have shown that under sample-limited conditions, traditional support vector machines (SVMs) can outperform deep models such as ResNet50 and YOLOv5 in classification performance [19]. Previous studies have also compared the performance of multiple deep neural network models. For example, in a classification task involving five common edible and toxic fungi in Thailand, AlexNet, ResNet50, and GoogLeNet were evaluated. The reported accuracies of all three models exceeded 95% on the dataset used in that study [20]. Beyond classic CNN architectures, graph-based image classification methods segment an image into semantically meaningful superpixels. This representation can substantially reduce computational cost while preserving spatial topology, which may offer advantages for mushroom classification [21]. In addition to improving network architectures and image representations, multi-feature fusion and visualization strategies have been reported to improve classification accuracy. For example, integrating feature maps generated by Gradient-weighted Class Activation Mapping (Grad-CAM), Local Interpretable Model-agnostic Explanations (LIME), and heatmap-based methods within a metaheuristic-optimized CNN can enhance the model’s ability to attend to discriminative regions and thereby improve overall performance [22]. At the deployment level, prior work combined transfer learning, a hybrid optimization algorithm (AHO), and a cyclical learning-rate schedule to build a high-accuracy mushroom classifier based on MobileNetV3. The study further demonstrated the feasibility and robustness of deployment on PCs, Android devices, and embedded edge platforms [23].

Overall, deep-learning-based machine vision methods show broad application potential because they enable rapid recognition, continuous operation, and, in some settings, accurate localization. However, their effectiveness often depends on large, high-quality training datasets. Most existing studies on edible mushroom classification use datasets with sufficient images and relatively balanced class distributions, and they pay less attention to performance under data-scarce or class-imbalanced conditions. To address this gap, this study constructed a small-sample dataset to evaluate the practical performance and robustness of different classification models under limited data and imbalanced class distributions. To address the challenge that fine-grained cues (e.g., gill texture and margin shape) can be lost during downsampling in conventional convolutional networks—particularly given the large number of mushroom species—this study proposes an improved model that incorporates an Adaptive Window Pyramid Fusion (AWPF) module. Without substantially increasing computational overhead, AWPF enhances multi-scale perception of fine-grained features and alleviates the classification bottleneck of conventional models when dealing with complex textures.

### Contributions

1. This study presents AWPF-ResNet18, an attention-fusion classifier based on ResNet18. The AWPF module integrates cross-scale attention with a feature pyramid to reduce semantic loss during downsampling, thereby enhancing fine-grained feature extraction for edible mushroom classification.
2. To handle large morphological variation and frequent occlusion, the proposed model uses a Dynamic Swin Window (DSW)-based variable-window strategy. The window size is adjusted across downsampling stages, enabling multi-scale capture and morphology-aware feature extraction. This design is well suited to edible mushroom classification, where inter-class shapes vary substantially while discriminative cues remain subtle.
3. This study constructs the Edible Mushroom Dataset by combining field-collected images with curated public data. The dataset reflects real-world challenges and supports robustness evaluation under resource-constrained conditions.

## Methodology

### AWPF-ResNet18

**Fig 1:**
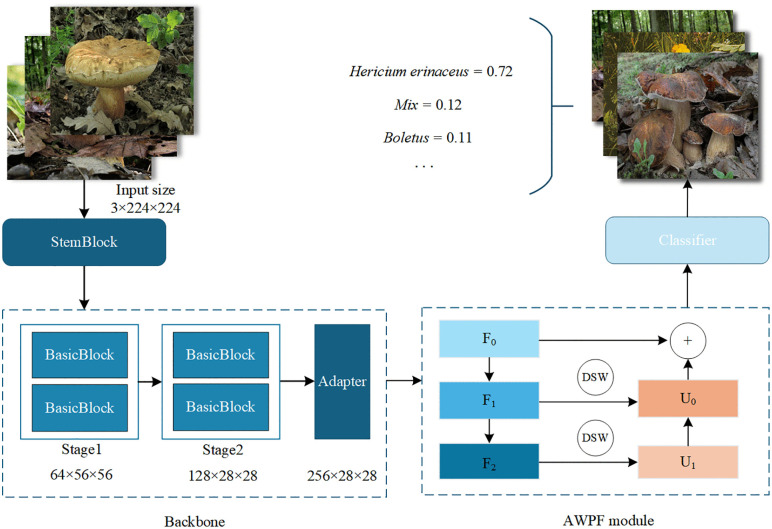
AWPF-ResNet18 Structure diagram. “StemBlock”: Initial Feature Extraction (7 × 7Conv ＆ 3 × 3MaxPool);“BasicBlock”: The fundamental residual unit of ResNet; “DSW”: Dynamic Swin Window module for window-based self-attention and local feature interaction. Reprinted from Mushroom Dataset (Roboflow Universe) under a CC BY 4.0 license, original copyright 2023 by Mushroom28. Source: https://universe.roboflow.com/mushroom28/mushroom-nksu4 (visited on 2025-12-30).

This study presents AWPF-ResNet18, a model architecture with ResNet18 as the backbone, designed to strengthen feature extraction for edible mushroom classification and improve recognition of subtle inter-class differences in complex backgrounds. In image classification, the backbone architecture is a key determinant of performance. As the core feature extractor, the backbone maps input RGB images to hierarchical representations that support downstream classification tasks. In this study, ResNet18 is adopted as the backbone for the classification task [24]. Compared with ResNet50 and deeper ResNet variants, ResNet18 offers a more lightweight architecture while retaining strong feature extraction capability. Using a lightweight backbone also helps isolate the contribution of the proposed module, reducing the likelihood that performance gains are attributed primarily to increased backbone depth. The overall architecture and proposed modifications are illustrated in Fig 1.

First, the feature extraction strategy of the backbone is optimized. The proposed model adopts ResNet18 as the backbone and applies a strategic truncation, using only the first two stages (Stage 1 and Stage 2) to extract base features. This design reduces the number of parameters and mitigates the loss of spatial details caused by excessive downsampling in deeper layers. To match the dimensional requirements of subsequent modules, a 1 × 1 convolutional adapter is appended to the backbone. It maps the feature channels to 256 and forms the initial feature pyramid $F_{\text{0}}$.

Second, an AWPF module is introduced to strengthen feature extraction. The module follows a “downsample–fuse–enhance” pipeline that mitigates information loss in conventional feature pyramids when modeling small-scale fungal cues. Unlike conventional Feature Pyramid Networks (FPNs) that rely mainly on convolution-based smoothing and fixed upsampling, AWPF incorporates cross-scale attention-based fusion to enable adaptive selection and aggregation of salient features [25]. In addition, a DSW is used to refine and concatenate local features, enabling hierarchical fusion across scales. This design allows the fusion process to adapt to feature maps at different resolutions, rather than using a fixed window configuration.

Finally, to accommodate scale variations of feature maps within AWPF, the proposed model introduces a DSW to improve the efficiency of local feature interactions. To alleviate boundary artifacts that may arise from fixed-size window partitioning, DSW dynamically adjusts the window size according to the feature-map resolution: 7 × 7 for the shallow feature $F_{\text{1}}$ and 5 × 5 for the deeper feature $F_{\text{2}}$. This design better matches window partitioning to the feature-map size and helps preserve small-scale fungal cues with less boundary loss. After multi-scale fusion and enhancement by AWPF, the resulting feature map is fed into the classification head. Global average pooling compresses the spatial dimensions into a feature vector, which is mapped to the class space by a linear layer to produce fine-grained edible mushroom predictions. By jointly optimizing backbone downsampling and the AWPF dynamic-window mechanism, the proposed model remains lightweight (<30M parameters) while improving edible mushroom recognition accuracy.

### Pseudocode of the proposed method

The following algorithm illustrates the data flow and feature processing procedure of the proposed network and describes its overall forward propagation process. The detailed structures of each module have been presented in the previous sections and are therefore not repeated here.

Input: Input image X; number of classes K

Output: Prediction logits y1:$F_{\text{0}}\leftarrow \text{ResNet18}\_\text{Backbone}(X)$

2:$F_{\text{1}}\leftarrow \text{Downsample}(F_{\text{0}})$

3:$F_{\text{2}}\leftarrow \text{Downsample}(F_{\text{1}})$

4:$U_{\text{1}}\leftarrow \text{DSW}(F_{\text{2}},\text{size}(F_{\text{1}}))$

5:$F_{\text{1},\text{enh}}\leftarrow F_{\text{1}}+U_{\text{1}}$

6:$U_{\text{0}}\leftarrow \text{DSW}(F_{\text{1},\text{enh}},\text{size}(F_{\text{0}}))$

7:$F_{\text{AWPF}}\leftarrow F_{\text{0}}+U_{\text{0}}$

8:$y\leftarrow \text{Head}(F_{\text{AWPF}})$

9: Return y

### AWPF module

**Fig 2:**
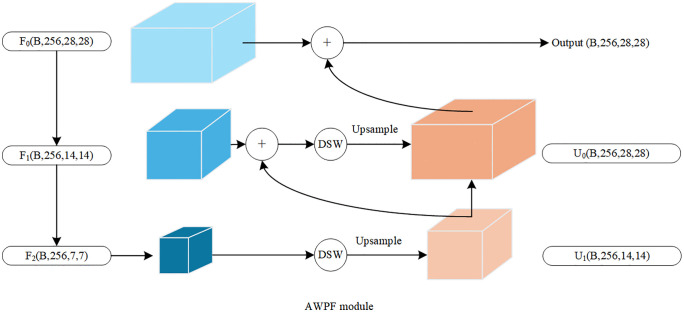
AWPF Structure diagram.

To address the challenges of subtle inter-class differences and complex texture patterns in edible mushroom classification, this study introduces an improved AWPF module. Unlike conventional FPNs, which typically rely on fixed upsampling and simple linear aggregation, the AWPF module adopts a convolution–attention co-design for feature fusion. Specifically, an embedded DSW module replaces the single convolutional smoothing operation used in FPNs. Using shifted-window self-attention, DSW enables broader-context interactions and dynamically recalibrates the fused features. This design mitigates information attenuation during feature propagation and helps the model attend to fine-grained texture cues that are critical for classification, as shown in Fig 2.

The module takes as input the feature map produced by ResNet18, with a spatial size of 28 × 28 and 256 channels. The AWPF module consists of three components: a downsampling pathway ($F_{\text{0}},F_{\text{1}},F_{\text{2}}$), a DSW layer, and an upsampling fusion pathway ($U_{\text{0}},U_{\text{1}}$). The downsampling pathway derives multi-scale feature maps from the backbone output while progressively reducing the spatial resolution. Let the input feature be $X\in {\mathbb{R}}^{B\times C\times 28\times 28}$. A top-down feature pyramid is first constructed using convolutional layers. Let $F_{i}$ denote the feature map at level i, which is computed as:


$$F_{i+1}=conv_{3\times 3}\left(F_{i},\text{s}=2,p=1\right),i\in \left\{0,1\right\}$$ (1)

Here, $conv_{3\times 3}\left(\cdot \right)$. denotes a 3 × 3 convolution with stride 2, followed by BN and a ReLU activation. This operation halves the spatial resolution and aggregates local information by enlarging the receptive field. To better model long-range dependencies beyond standard convolutions, a DSW module is introduced at each fusion node. The enhancement and fusion follow a bottom-up pathway: the deeper feature $F_{i+1}$ is upsampled and fused with the shallower feature $F_{i}$. The fused feature at level i, denoted as $U_{i}$, is computed as:


$$U_{i}=DWS\left(F_{i+1}+Upsample\left(U_{i+1}\right)\right)$$ (2)

Specifically, for the deepest feature $F_{\text{2}}$, DSW is applied directly as the starting point of the fusion process:


$$U_{\text{1}}=DWS\left(Upsample\left(F_{\text{2}}\right)\right)$$ (3)

In this equation, Upsample(·) denotes bilinear interpolation used to align spatial dimensions, and “+” denotes element-wise addition for feature fusion. The resulting output feature map is $U_{\text{0}}$. This design facilitates the propagation of high-level semantic information to guide the extraction of shallow texture cues. With self-attention in DSW, the model can adaptively emphasize discriminative regions, such as cap texture patterns.

### Dynamic Swin window module

**Fig 3:**
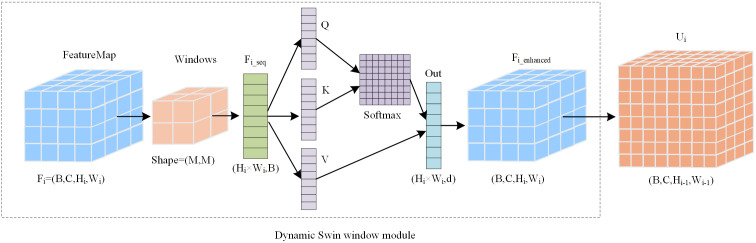
Dynamic Swin window Structure diagram.

To alleviate boundary artifacts that may occur when FPNs process feature maps with non-standard sizes, the proposed model further adopts an adaptive window strategy that adjusts the window size according to the resolution of each pyramid level, as shown in Fig 3. This design addresses the limitation that conventional FPNs use a fixed receptive field that cannot adapt to feature content, and it improves discrimination of cap-texture and stipe-pattern cues under complex backgrounds [26,27].

The module receives a pyramid feature map, extracts window-based global features using DSW, upsamples the result to match the spatial size of the previous level, and outputs $U_{i}$ for subsequent cross-scale fusion. Let the input feature be $F_{i}\in {\mathbb{R}}^{B\times C\times H_{i}\times W_{i}}$, where B is the batch size, C is the number of channels, and $H_{i}$ and $W_{i}$ are the height and width of the feature map, respectively. Given a window size M on the input feature map, the window partitioning can be expressed as:


$$\begin{array}{c} Size\left(M_{i}\right)=\left\{ \begin{array}{c} @l@\begin{array}{cc} 7 & i=1 \end{array}\\ \begin{array}{cc} 5 & i=2 \end{array} \end{array}\right. \end{array}$$ (4)

This adaptive mechanism prevents windows from exceeding the feature-map boundaries and is suitable for small feature maps in the pyramid. Two window sizes (7 × 7 and 5 × 5) are used to process feature maps at different scales. The window-shifting strategy is defined as follows:


$$\begin{array}{c} Shift\left(M_{i}\right)=\left\{ \begin{array}{cc} \text{0} & \begin{array}{cc} if & i=\text{0}(\text{mod}\text{2}) \end{array}\\ \text{min}(\text{3},\left\lfloor \frac{H_{i}}{\text{2}}\right\rfloor ,\left\lfloor \frac{W_{i}}{\text{2}}\right\rfloor ) & otherwise \end{array}\right. \end{array}$$ (5)

When i is even, standard (non-shifted) windows are used; when i is odd, the windows are shifted according to the strategy above. This alternating scheme enables information exchange across neighboring windows through shifted partitioning. The spatial features are then reshaped into a sequence, where $N_{i}=H_{i}\times W_{i}$ is the total number of spatial positions. Each spatial position is represented as an individual feature vector, forming a sequence of length $N_{i}$, which can be written as:


$$F_{i\_seq}\text{\textbackslash{} }=\text{\textbackslash{} flatten}{\left(F_{i},\left[B,H_{i}\times W_{i}\right]\right)}^{\text{T}}$$ (6)

The within-window attention computation is defined as follows:


$$\text{Attention}\left(Q,K,V\right)=Softmax\left(\frac{{\text{QK}}^{\text{T}}}{{\sqrt{\text{d}}}_{\text{k}}}+B\right)V$$ (7)

Let Q, K, and V denote the query, key, and value matrices, respectively, and let B denote the bias term. The attention weights are computed from the scaled similarity between Q and K, normalized by Softmax, and then used to aggregate V. The bias B and the scaling factor $1/\sqrt{d_{k}}$ help stabilize training and enable dynamic feature refinement within each window. Together, these operations form the within-window self-attention used in DSW. After several DSW blocks, the sequence representation is reshaped back to a feature-map format and upsampled to a matched spatial size for subsequent fusion, which can be written as:


$$F_{i\_enhanced}=reshape\left(transpose\left(Out\right),\left[B,C,H_{i},W_{i}\right]\right)$$ (8)

$$U_{i}=Upsample\left(F_{i\_enhanced},\left(H_{i-1},W_{i-1}\right)\right)$$ (9)

## Experiment and result

### Dataset

**Fig 4:**
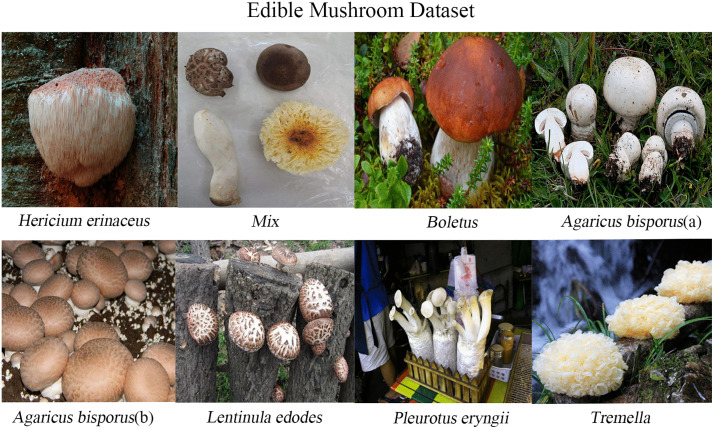
Edible Mushroom dataset. “Agaricus bisporus”has two morphologies:one is milky white (a),and the other is brown(b).To ensure academic rigor in nomenclature,all categories in this study are labeled using their Latin scientific names. Reprinted from Mushroom Dataset (Roboflow Universe) under a CC BY 4.0 license, original copyright 2023 by Mushroom28. Source: https://universe.roboflow.com/mushroom28/mushroom-nksu4 (visited on 2025-12-30).

The experiments use the Edible Mushroom Dataset in Fig 4, which is designed for few-shot image classification. The “Mix” class consists of field-collected images in which the mushroom morphologies correspond to species that also appear as individual classes. This class is intended to simulate real-world scenarios where multiple edible mushrooms are placed together. The remaining six single-species classes are derived from public datasets available on Roboflow; only images meeting the study requirements were selected from the original sources. The dataset provides a clear representation of morphological variation across edible mushroom species. In total, the dataset contains 2,435 images. For each class, images are split into training and validation sets with a 7:3 ratio. Table 1 reports the number of images per class in the training and validation sets.

**Table 1:** Edible Mushroom Dataset.

Category	Train(n)	Validation(n)
*Hericium erinaceus*	304	108
*Mix*	14	10
*Boletus*	233	106
*Agaricus bisporus*	187	71
*Lentinula edodes*	446	174
*Pleurotus eryngii*	283	148
*Tremella*	246	105

**Note:**The above table shows the number of samples used for training and validation for each type of edible mushroom. Here,“Mix” indicates that this category contains samples from any of the other six categories.

### Experimental environment setup

To evaluate the proposed AWPF-ResNet18 model, all experiments are conducted under the same hardware platform and software framework to improve fairness and reproducibility. The experimental environment and hyperparameter settings are summarized in Table 2 and Table 3.

**Table 2:** Experimental environment of deep learning.

Configuration Environment	Configuration Name
Operating System	Windows10
CPU	13th Gen Intel(R)Core(TM) i7-13700HX
GPU	RTX 4080Laptop(12GB)×1
Deep learning framework	PyTorch 2.3.1
Compiler	Python 3.9.0
Acceleration module	CUDA Toolkit-11.8

**Table 3:** Experimental hyperparameters of deep learning.

Parameter	Numerical value
Image size	224 × 224
Batch size	32
Learning Rate	0.001
Optimizer	AdamW
Epochs	50

### Evaluation metrics

During the research, it was found that the primary challenges in few-shot classification lie in three aspects: insufficient data, poor diversity, and uneven sample distribution. To quantitatively evaluate the classification performance of the network model on the “Edible Mushroom Dataset”, accuracy (Acc), macro precision (MP), macro F1-score (MF), and macro recall (MR) were adopted as comprehensive evaluation metrics. Suppose there are *k* different categories with a total of n samples, where *TPᵢ* represents the number of samples belonging to class *i* and correctly predicted as class *i*, *FNᵢ* represents the number of samples belonging to class *i* but incorrectly predicted as other classes, *FPᵢ* represents the number of samples not belonging to class *i* but incorrectly predicted as class *i*, and *TNᵢ* represents the number of samples not belonging to class *i* and correctly predicted as other classes. The expressions of the above four parameters can be represented as:


$$\text{\textbackslash{} }Acc=\frac{\overset{k}{\underset{i=1}{\sum }}TP_{i}}{n}$$ (10)

$$MP=\text{\textbackslash{} }\frac{\text{1}}{k}\overset{k}{\underset{i=1}{\sum }}\frac{TP_{i}}{TP_{i}+FP_{i}}$$ (11)

$$MR=\text{\textbackslash{} }\frac{\text{1}}{k}\overset{k}{\underset{i=1}{\sum }}\frac{TP_{i}}{TP_{i}+FN_{i}}$$ (12)

$$MF=\text{\textbackslash{} }\frac{\text{1}}{k}\overset{k}{\underset{i=1}{\sum }}\frac{2TP_{i}}{2TP_{i}+FP_{i}+FN_{i}}$$ (13)

Among the above four evaluation metrics, MR can reflect the ability of the model to identify samples of a certain class. Meanwhile, its calculation method-whereby the recall for each class is computed first, followed by taking the arithmetic mean of these recall rates-can disregard differences in the number of samples across classes, ensuring that the recall rate of each class carries equal weight in the final result. For this reason, greater emphasis is placed on this metric.

### Experiment results

To assess classification accuracy on the Edible Mushroom Dataset, the proposed method is compared with nine commonly used classifiers under the same hyperparameter settings. To improve the robustness of the evaluation, each model is trained and tested using five different random seeds, and the results are reported as mean ± standard deviation in Table 4.

**Table 4:** Val-Performance.

Model	Acc(%)	MP(%)	MF(%)	MR(%)	P	AccTop-1 Image-1K (%)
ResMLP-12	90.9 ± 0.4	87.1 ± 0.1	92.0 ± 0.2	92.3 ± 0.3	15M	77.1
ResNet18	90.7 ± 0.1	86.7 ± 0.2	88.9 ± 0.3	91.6 ± 0.2	11.7M	69.8
RegNet	92.2 ± 0.3	93.3 ± 0.4	91.9 ± 0.4	92.9 ± 0.1	4.3M	74.1
LeViT-128S	92.6 ± 0.4	92.3 ± 0.2	92.4 ± 0.5	93.1 ± 0.3	7M	76.6
MobileNetV3-L	92.4 ± 0.2	92.6 ± 0.3	92.8 ± 0.2	93.1 ± 0.4	5.4M	75.2
ShuffleNetV2	92.5 ± 0.1	91.2 ± 0.1	91.7 ± 0.1	92.8 ± 0.2	1.9M	72.6
ConvNeXt-T	**93.5** ± 0.4	88.2 ± 0.5	90.2 ± 0.3	93.3 ± 0.1	28M	82.1
Swin-T	87.8 ± 0.6	79.4 ± 0.3	81.5 ± 0.1	87.8 ± 0.2	28.3M	81.3
Swin-B	90.5 ± 0.3	86.1 ± 0.3	86.3 ± 0.2	89.5 ± 0.5	87.8M	83.5
VIT-16	24.8 ± 0.4	37.6 ± 0.1	21.4 ± 0.3	36.8 ± 0.2	85.7M	78
AWPF-ResNet18	93.2 ± 0.2	**94.2** ± 0.2	**93.9** ± 0.3	**93.6** ± 0.1	29M	–

**Note:**The best results in each column are highlighted in bold.

As shown in Table 4, the proposed model achieves the best overall performance across MP, MF, and MR, with consistent gains over the compared baselines. The mean performance gap across metrics is within 0.33 percentage points. These results suggest that, for few-shot agricultural image classification in this study, combining convolutional inductive biases with attention can be advantageous compared with purely MLP-based designs. The proposed model is 0.3 percentage points below ConvNeXt-T in accuracy. This small gap is expected, as ConvNeXt-T is a high-capacity CNN architecture optimized for large-scale benchmarks (e.g., ImageNet-1K), which can yield stronger representations and higher accuracy. In addition, AWPF-ResNet18 improves MP by 0.9 percentage points over the second-best RegNet. This improvement may indicate that AWPF’s multi-scale fusion is effective at capturing subtle discriminative cues in edible mushroom images under the studied conditions.

**Fig 5:**
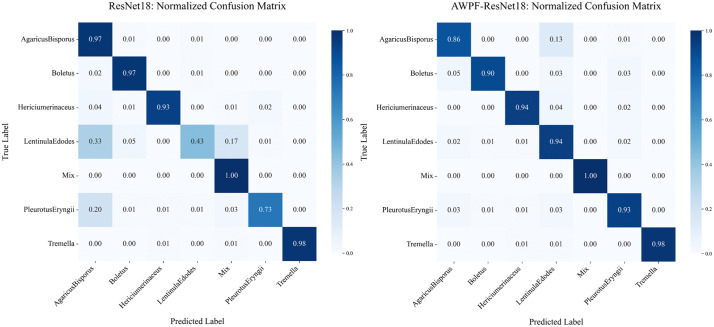
Normalized confusion matrices comparing the classification performance of different models. (a)Confusion matrix of the base model,(b)Confusion matrix of the model with the AWPF module added.

**Fig 6:**
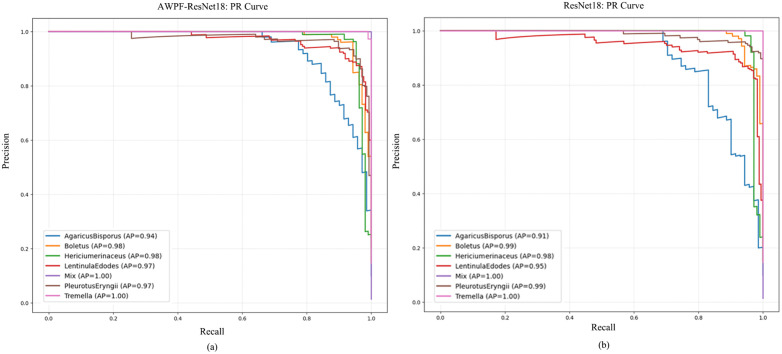
Precision-Recall(PR)curves comparing the performance of different models. (a) PR plot of the model with the AWPF module added, (b) PR plot of the base model.

The results also indicate that performance on ImageNet-1K is not strictly correlated with performance on the test set used in this study. One plausible reason is the class-imbalanced distribution of the constructed dataset. When training data are limited, overly complex models are more prone to overfitting. This tendency is more pronounced for Transformer-based models in the present experiments, with ViT-16 showing the largest degradation. For lightweight models suitable for mobile deployment (e.g., MobileNetV3-L and ShuffleNetV2), the metrics are more balanced, suggesting good suitability for few-shot classification under the studied setting. The confusion matrix in Fig 5 shows that the baseline ResNet18 has difficulty distinguishing visually similar species. In particular, the brown variant of *Agaricus bisporus* is frequently confused with *Lentinula edodes*, likely because their cap color and texture are similar. After incorporating AWPF, recall for *Lentinula edodes* increases by 51%. This improvement suggests that AWPF captures cross-scale information spanning coarse morphology and fine details, thereby improving discrimination among morphologically similar edible mushrooms. In addition, the PR curves in Fig 6 provide further evidence of the model’s overall robustness. After incorporating AWPF, the area under the PR curve increases across classes, and the curves shift toward the upper-right region. These trends suggest improved precision–recall trade-offs not only for hard classes but also at the overall level.

## Discussion

### Evaluation with different attention modules

To evaluate how different attention modules improve the baseline ResNet18, this study examines the interaction between cross-scale fusion and window strategies. The results are summarized in Table 5.

**Table 5:** Comparative experiment.

Model	Acc	MP	MF	MR
ConvNeXt-T(base)	**93.5%**	88.2%	90.2%	93.3%
ResNet18	90.7%	86.7%	88.9%	91.6%
ResNet18 + Swin	92.2%	91.5%	91.1%	92.7%
ResNet18 + SE	91.7%	92.2%	90.8%	86.2%
ResNet18 + CBAM	89.3%	90.1%	86.8%	87.7%
ResNet18 + AWPF	93.2%	**94.2%**	**93.9%**	**93.6%**
ResNet18 + AWPF+SE	90.1%	90.9%	83.5%	89.8%

As shown in Table 5, ConvNeXt-T is selected as the baseline due to its strong performance with a comparable parameter budget, and several mainstream attention modules are added for comparative evaluation. The results show that ConvNeXt-T achieves strong performance even without explicit attention modules, suggesting that attention is not the only route to improved accuracy. Overall, models that combine CNN components with Transformer-style self-attention tend to outperform purely CNN-based designs. For example, the hybrid model equipped with window-based self-attention outperforms variants that use convolutional attention modules such as SE or CBAM across evaluation metrics. This trend is more evident for the proposed AWPF design, which yields larger gains in this ablation setting. Notably, adding SE reduces MR by 6.5 percentage points. One possible explanation is that SE overemphasizes a subset of channels, which suppresses other informative channels and leads to less complete representations, thereby reducing recognition for certain classes.

In addition, stacking multiple attention modules can degrade performance. For instance, combining AWPF with SE results in lower overall performance than the baseline, with MF showing the largest drop (5.4 percentage points). In the proposed network, AWPF forms a CNN–self-attention hybrid that aims to retain strong representation capacity while reducing the parameter and compute overhead typically associated with Transformer-style designs, and it also compensates for the limited global-context modeling of standard convolutions. This multi-scale mechanism supports learning features at different spatial scales. On the Edible Mushroom Dataset, the proposed method improves MP, MF, and MR by 6.0, 3.7, and 0.3 percentage points, respectively, relative to ConvNeXt-T.

### Ablation study

To more accurately assess the contribution of each component in AWPF, a systematic ablation study is conducted along two dimensions: cross-scale fusion and window strategy. The results are reported in Table 6.

**Table 6:** Ablation experiment.

Cross-Scale	Win-Mix	Win（5）	Win（7）	Acc	MR
		√		90.7% 88.7%	91.6% 84.1%
			√	86.2%	84.0%
√	√			89.5% 91.5%	85.5% 92.6%
√		√		92.5%	91.0%
√ √	√		√	91.2% 93.2%	92.1% 93.6%

Here, “Cross-Scale” indicates the use of cross-scale fusion; “Win-Mix” denotes variable-size sliding windows; “Win (5)” and “Win (7)” denote fixed 5 × 5 and 7 × 7 windows, respectively. To systematically evaluate the contribution of components in AWPF-ResNet18, this study examines the interaction between cross-scale fusion and window strategies. The results suggest that Cross-Scale alone serves as a semantic anchor: by aggregating global context, it provides stable gains and supports the fusion of local and global features. In addition, the fixed 5 × 5 setting achieves 88.7% accuracy, outperforming the larger 7 × 7 window. A plausible explanation is that although a 7 × 7 window offers a larger receptive field in shallow layers, applying it to deeper layers can require unnecessary padding due to size mismatch. Such padding may introduce boundary artifacts and background noise, weakening high-level semantic features. While Cross-Scale can partially mitigate this degradation by providing more stable representations, it may not fully compensate for the structured noise introduced by padding. By contrast, the proposed Win-Mix strategy assigns 7 × 7 windows to shallow layers and 5 × 5 windows to deeper layers, improving alignment across scales. Notably, some ablation variants perform worse than the full ResNet18 reported in Table 6. This outcome is expected because a truncated backbone is used (Stages 3 and 4 are removed) to isolate the effect of AWPF under a controlled setting.

### Visual explanation analysis

**Fig 7:**
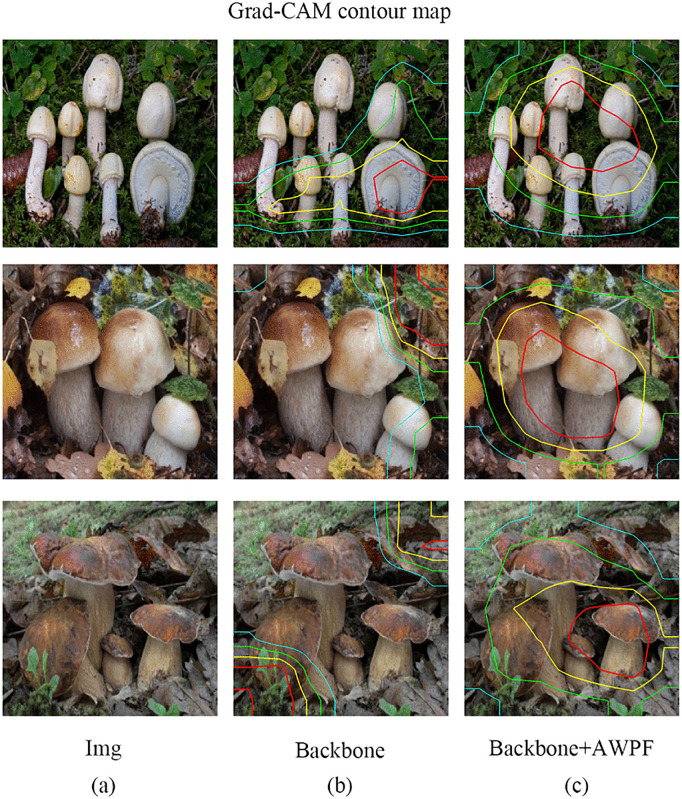
Grad-CAM maps of the backbone and AWPF model. (a) Original images; (b) backbone model; (c) backbone model with the proposed AWPF module. Reprinted from Mushroom Dataset (Roboflow Universe) under a CC BY 4.0 license, original copyright 2023 by Mushroom28. Source: https://universe.roboflow.com/mushroom28/mushroom-nksu4 (visited on 2025-12-30).

**Fig 8:**
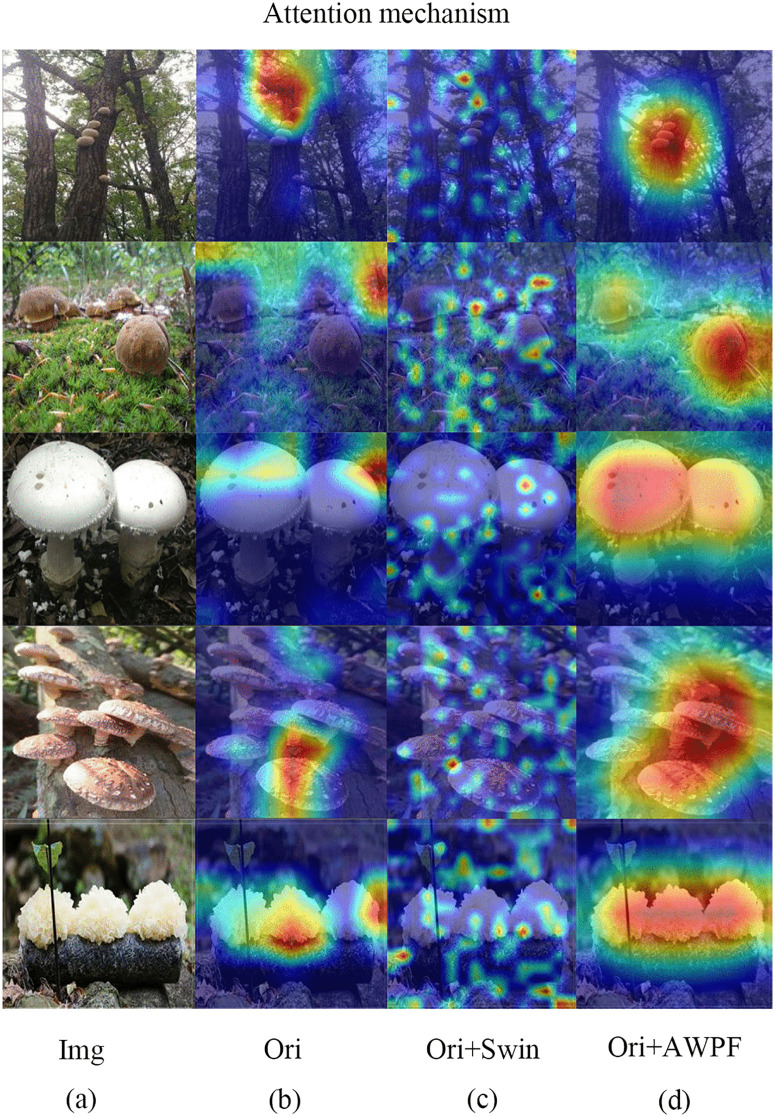
Heatmap comparison between the baseline and improved models. (a) Original images; (b) baseline model; (c) model with the Swin Transform module; (d) model with the proposed AWPF module. Reprinted from Mushroom Dataset (Roboflow Universe) under a CC BY 4.0 license, original copyright 2023 by Mushroom28. Source: https://universe.roboflow.com/mushroom28/mushroom-nksu4 (visited on 2025-12-30).

Contour maps and heatmaps provide intuitive visualizations of the regions attended by the model during feature extraction, thereby facilitating the analysis of attention distribution characteristics in the outputs of different modules. As shown in Fig 7, the Grad-CAM contour maps of the proposed model are generated for both the backbone network and the network enhanced with the AWPF module. Compared with the backbone network, the model incorporating AWPF forms more compact response regions that are more consistent with the target areas, indicating that AWPF not only strengthens the model’s attention to foreground regions but also improves the spatial localization of discriminative mushroom regions. Furthermore, as shown in Fig 8, compared with the model equipped with the Swin module, AWPF allocates more attention to the mushroom regions, thereby enhancing feature extraction for the target areas and exhibiting stronger foreground-focused capability than the baseline network. These visualizations suggest that incorporating AWPF into the overall architecture helps improve target-oriented feature learning.

## Conclusions

This study proposes an attention-fusion classification model, AWPF-ResNet18, which enhances the global attention capability across downsampling layers by incorporating the AWPF attention module into the base ResNet18. The model demonstrates superior performance in classification tasks involving targets with significant size variations. To validate the superiority of the proposed model, This study constructed a dataset of edible mushrooms consisting of 2,435 images across seven different categories. Comparative experiments with eight other classification models on this dataset confirmed the effectiveness of the AWPF module. With only a slight increase in model parameters, the proposed model achieves optimal performance among models with comparable parameter counts and shows varying degrees of improvement over other models in the MP, MF, and MR metrics. The constructed attention heatmaps also illustrate that the AWPF module focuses more effectively on the main regions of the image compared to other similar modules. In the future, we plan to further reduce the overall parameter count of the model and explore its potential applications in other classification domains.
